# Functional Activities Detected in the Olfactory Bulb and Associated Olfactory Regions in the Human Brain Using T2-Prepared BOLD Functional MRI at 7T

**DOI:** 10.3389/fnins.2021.723441

**Published:** 2021-09-13

**Authors:** Xinyuan Miao, Adrian G. Paez, Suraj Rajan, Di Cao, Dapeng Liu, Alex Y. Pantelyat, Liana I. Rosenthal, Peter C. M. van Zijl, Susan S. Bassett, David M. Yousem, Vidyulata Kamath, Jun Hua

**Affiliations:** ^1^Neurosection, Division of MRI Research, Russell H. Morgan Department of Radiology and Radiological Science, School of Medicine, Johns Hopkins University, Baltimore, MD, United States; ^2^F.M. Kirby Research Center for Functional Brain Imaging, Kennedy Krieger Institute, Baltimore, MD, United States; ^3^Department of Neurology, School of Medicine, Johns Hopkins University, Baltimore, MD, United States; ^4^Department of Biomedical Engineering, Johns Hopkins University, Baltimore, MD, United States; ^5^Department of Psychiatry and Behavioral Sciences, School of Medicine, Johns Hopkins University, Baltimore, MD, United States; ^6^Department of Radiology, Johns Hopkins Hospital, Baltimore, MD, United States

**Keywords:** olfaction, habituation, MRI artifacts, T2prep, high-field

## Abstract

Olfaction is a fundamental sense that plays a vital role in daily life in humans, and can be altered in neuropsychiatric and neurodegenerative diseases. Blood oxygenation level-dependent (BOLD) functional magnetic resonance imaging (fMRI) using conventional echo-planar-imaging (EPI) based sequences can be challenging in brain regions important for olfactory processing, such as the olfactory bulb (OB) and orbitofrontal cortex, mainly due to the signal dropout and distortion artifacts caused by large susceptibility effects from the sinonasal cavity and temporal bone. To date, few studies have demonstrated successful fMRI in the OB in humans. T2-prepared (T2prep) BOLD fMRI is an alternative approach developed especially for performing fMRI in regions affected by large susceptibility artifacts. The purpose of this technical study is to evaluate T2prep BOLD fMRI for olfactory functional experiments in humans. Olfactory fMRI scans were performed on 7T in 14 healthy participants. T2prep BOLD showed greater sensitivity than GRE EPI BOLD in the OB, orbitofrontal cortex and the temporal pole. Functional activation was detected using T2prep BOLD in the OB and associated olfactory regions. Habituation effects and a bi-phasic pattern of fMRI signal changes during olfactory stimulation were observed in all regions. Both positively and negatively activated regions were observed during olfactory stimulation. These signal characteristics are generally consistent with literature and showed a good intra-subject reproducibility comparable to previous human BOLD fMRI studies. In conclusion, the methodology demonstrated in this study holds promise for future olfactory fMRI studies in the OB and other brain regions that suffer from large susceptibility artifacts.

## Introduction

The sense of smell has a profound yet underappreciated influence on physical and mental wellbeing. Olfactory information aids the detection of spoiled food and environmental hazards and influences personal hygiene and feeding behavior. Odorants are received by olfactory sensory neurons embedded in the nasal epithelium (Buck and Axel, 1991), which project first to glomeruli within the olfactory bulbs (OBs) and then to subcortical and cortical targets. Olfactory deficits have emerged as an early risk factor in many neurodegenerative and neurodevelopmental conditions (Moberg et al., 2014; Fullard et al., 2017; Marin et al., 2018), as well as in healthy older adults (Doty and Kamath, 2014). Olfactory disturbance is also a common symptom in coronavirus disease (COVID) infection (Moein et al., 2020).

Blood oxygenation level-dependent (BOLD) functional magnetic resonance imaging (fMRI) has been widely used to investigate functional activities in brain regions associated with olfaction. Functional activations during olfactory stimulation have been detected by BOLD fMRI in the primary olfactory (piriform) cortex and many secondary olfactory regions in the human brain (Yang et al., 1997; Sobel et al., 1998, 2000; Poellinger et al., 2001; Gottfried et al., 2002). However, several olfactory related regions such as the OB and orbitofrontal cortex are difficult to image with the conventional gradient echo (GRE) echo-planar-imaging (EPI) based BOLD fMRI methods, mainly due to the well-known signal dropout and distortion artifacts caused by large susceptibility effects from the nearby sinonasal cavity and temporal bone especially the aerated petrous apex (Yang et al., 1997; Sobel et al., 1998, 2000; Poellinger et al., 2001; Wang et al., 2010; Zong et al., 2014; Lu et al., 2018). Such susceptibility artifacts in olfactory regions have been reported at 3.0 Tesla (3T), which is currently the most commonly used field strength for clinical MRI, and lower fields (Yang et al., 1997; Sobel et al., 1998, 2000; Poellinger et al., 2001; Wang et al., 2010; Lu et al., 2018); and are exacerbated at higher magnetic fields such as 7.0 Tesla (7T). It is especially challenging to do fMRI in the OB because of significant susceptibility artifacts and the small size of the bulb. Functional activities in the OB have been reported in rodents using fMRI (Xu et al., 2000, 2003, 2005; Schafer et al., 2005; Martin et al., 2007; Li et al., 2014; Poplawsky and Kim, 2014; Murphy et al., 2016; Zhao et al., 2016, 2017; Muir et al., 2019) and manganese enhanced MRI (Cross et al., 2004), in dogs (Jia et al., 2014; Berns et al., 2015) using fMRI, and in non-human primates using fMRI (Boyett-Anderson et al., 2003; Zhao et al., 2015). A recent human study used a surface coil covering most of the primary olfactory regions to do fMRI in the OB (Fournel et al., 2020). The electrobulbogram (EBG) technique has been developed recently for non-invasive recording of functional signals in the human OB (Iravani et al., 2020). To date, however, few studies have reported successful fMRI in the OB in humans with whole brain coverage. As olfactory stimulation can elicit functional activities in a variety of brain regions along the olfactory pathways (Doty, 2015), a whole brain coverage will be ideal for many olfactory fMRI studies in humans.

Recently, a whole-brain T2-prepared (T2prep) BOLD fMRI (Hua et al., 2014) approach showed minimal susceptibility artifacts across the entire brain in healthy subjects. In the T2prep BOLD approach (Supplementary Figure S1), the BOLD contrast is induced using driven equilibrium (Becker, and Farrar, 1969), also referred to as a T2 preparation or T2-prep (Haase, 1990; Parrish and Hu, 1994) module, followed by a single-shot 3D fast GRE readout, which is commonly used in high-resolution anatomical scans with much reduced susceptibility artifacts compared to EPI. The T2-prep BOLD method minimized the susceptibility artifacts and enhanced functional sensitivity in the brains of individuals with metallic head implants (Miao et al., 2020), and in brain regions close to blood products and/or calcifications in patients undergoing presurgical brain mapping (Hua et al., 2017; Miao et al., 2020). In the current study, we tested the performance of T2prep BOLD fMRI in olfactory functional experiments in healthy subjects conducted on a 7T human MRI system. The contrast-to-noise ratio (CNR) was compared between T2prep BOLD fMRI and conventional GRE EPI BOLD fMRI in the same subjects. Functional activation in olfactory-eloquent brain regions, especially the OB, was assessed using T2prep BOLD fMRI. The reproducibility of the T2prep BOLD fMRI results was also evaluated. The primary goal of this technical study is to evaluate the feasibility and reproducibility of performing olfactory fMRI using T2prep BOLD fMRI in healthy human subjects. A comprehensive assessment and validation of the underlying neuronal and physiological origins of the fMRI signal changes is beyond the scope of the current study and is being pursued in subsequent studies.

## Materials and Methods

### Participants

Fourteen healthy participants (47 ± 11 yo, 8 females) were recruited in the study. Some experiments such as GRE EPI BOLD fMRI scans during olfactory stimulation (see section “MRI Scans”) was performed in a subset of participants (*n* = 5, see power analysis in section “Statistical Analysis”) for comparison. We declare that all experiments on human subjects were conducted in accordance with the Declaration of Helsinki. This study was approved by the Johns Hopkins Institutional Review Board, and written informed consent was obtained from each participant. Participants had no history of neurologic or psychiatric disorders, or history of sinus surgery, craniofacial abnormalities, or nasal trauma or surgery. All participants were right-handed, non-smokers, and were not on any medications. Individuals experiencing respiratory infection, sinus allergies or symptoms of a common cold within a month before the study visit were excluded. The University of Pennsylvania Smell Identification Test (UPSIT; Doty et al., 1984, 1989) was administrated to all participants, and the results indicated that all participants had normal olfactory functions.

### Magnetic Resonance Imaging Scans

All scans were performed on a 7.0 Tesla (7T) Philips MRI scanner (Philips Healthcare, Best, Netherlands). An 8-channel transmit head coil was used for signal transmission and a 32-channel phased array head coil was used for signal reception. An advanced B0 shim algorithm was applied using the MRCodeTool software (v1.5.9, TeslaDC, Zaltbommel, Netherlands) installed on the scanner to improve B0 field homogeneity over the entire brain. In order to improve B1 field homogeneity, rectangular pads filled with high dielectric constant materials (Teeuwisse et al., 2012) were placed on the side of the subjects’ head. The breathing pattern was recorded for each participant using a respiratory belt placed around the participant’s abdomen so that it can be regressed out for fMRI analysis.

The following scans were performed for each participant:

(1) 3D T1-weighted Magnetization Prepared RApid Gradient Echo (MPRAGE): repetition time (TR)/inversion time (TI)/echo time (TE) = 4500/563/1.81 ms; field of view (FOV) = 220 mm × 220 mm; voxel = 1 mm isotropic; 180 sagittal slices;
(2) T2prep BOLD fMRI during the olfactory paradigm described next: TR = 2000 ms; flip angle = 4°; T2prep effective TE = 50 ms; FOV = 180 (RL) × 222 (AP) mm^2^; voxel = 1.5 mm isotropic; 84 axial slices covering the entire brain; parallel imaging with SENSE factor = 3 × 3 (AP × FH), single-shot 3D turbo field echo (3D TFE, also known as 3D fast GRE) readout, centric phase encoding profile starting from the center of k-space, TR_*GRE*_/TE_*GRE*_ = 2.90/1.32 ms. A first-order volume shim was performed on the imaging volume, which is usually sufficient for improving field homogeneity as shown in previous studies (Hua et al., 2014; Miao et al., 2020).
  The following scan was performed in a subset of participants (*n* = 5, see power analysis in section “Statistical Analysis”) on a different day (in order to avoid olfactory habituation effects) to compare the CNR between GRE EPI BOLD and T2prep BOLD fMRI:
(3) GRE EPI BOLD fMRI during the same olfactory paradigm: TR = 2000 ms; flip angle = 70° (approximately the Ernst angle); TE = 22 ms; FOV = 180 (RL) × 222 (AP) mm^2^; voxel = 1.5 mm isotropic; 33 axial slices; SENSE = 3 (AP), single-shot 2D GRE EPI readout. Note that the coverage of conventional 2D GRE EPI scan was reduced as our current settings on 7T cannot achieve whole brain coverage with the chosen resolution, but the spatial and temporal resolutions were kept identical for the comparison. An optimal high-order shim method was performed on the imaging volume of the GRE EPI scan to improve field homogeneity and reduce dropout and distortion, and a field map based method was employed for distortion correction in GRE EPI (Schar et al., 2004).

### Olfactory Paradigm

**FIGURE 1:**
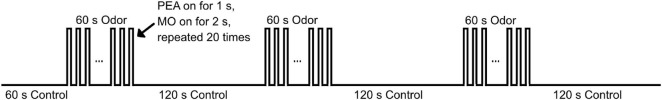
Illustration of the olfactory paradigm used during fMRI. The odorant during the 60 s stimulus-on block was delivered in a pulsed pattern, with 1 s odorant of PEA and 2 s of odorless mineral oil (MO) repeated for 20 times. During the 120 s stimulus-off block, only MO was delivered continuously.

An enhanced model of multi-channel computer-controlled olfactometer (Lundström et al., 2010) (Whiff LLC, Swarthmore, PA, United States) was used to deliver the odorants in precisely timed pulses. The olfactometer was placed outside of the scanning room and was connected to pressured air tanks. Phenyl ethyl alcohol (PEA, Sigma-Aldrich) diluted in odorless mineral oil (50% v/v, 60 ml) was embedded in a constantly flowing humidified air stream (1.5 L per min/nostril) at body temperature. PEA is a relatively pure olfactory nerve stimulant with relatively low trigeminal stimulation properties (Doty et al., 1978, 1984, 1986). The odorants was presented to both nostrils using a nasal cannula (Teleflex Medical) connected to the olfactometer via Everbilt vinyl tubing (inner diameter: 0.25 inch). The olfactory paradigm (Figure 1) started with a stimulus-off period of 60 s with odorless mineral oil, followed by three blocks of a stimulus-on period of 60 s with PEA and a stimulus-off period of 120 s with odorless mineral oil (total duration 10 min). The relatively long stimulation period was chosen in this study to evaluate habituation effects in the olfactory system. During the stimulus-on periods, the PEA was delivered in a pulsed pattern with 20 repetitions of 1 s PEA and 2 s odorless mineral oil, similar to previous studies (Welge-Lussen et al., 2009; Georgiopoulos et al., 2018). All participants were instructed to breathe passively through the nose and avoid sniffing. In addition, all participants were instructed to press a button box when they start to smell the PEA odor at the beginning of the 60 s stimulus-on period. If the button press is delayed for more than 3 s from the actual onset of the stimulus, the data is discarded and the participant is scheduled for another experiment on a different day (to avoid any potential habituation effects).

### Data Analysis

**FIGURE 2:**
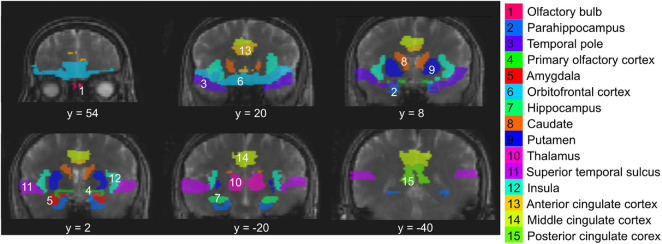
Regions-of-interest (ROI) evaluated in the study overlaid on T2prep BOLD fMRI images from one subject. These include the olfactory bulb (#1), primary olfactory cortex (#4, piriform cortex), and other secondary olfactory regions known from the literature. Note that only six coronal slices were provided to illustrate the locations of the ROIs, but many ROIs can cover more slices. Most olfactory regions are best viewed in the coronal plane.

Data analysis was performed mainly using the statistical parametric mapping (SPM) software package (Version 12, Wellcome Trust Centre for Neuroimaging, London, United Kingdom) and in-house routines coded in MATLAB R2019a (MathWorks, Natick, MA, United States). Realignment was performed for all fMRI images to correct for subject motion during the scans. Spatial smoothing was performed for fMRI images using an isotropic Gaussian kernel of 4 mm. The baseline drift of fMRI time series was removed by applying a high-pass filter with a cut-off frequency of 1/180 Hz (as the duration of one block in the olfactory paradigm is 180s) using the FMRIB Software Library (FSL6.0.1; FMRIB, Oxford, United Kingdom). An independent component analysis (ICA) based denoising approach (Salimi-Khorshidi et al., 2014) was performed on the fMRI data using FSL6.0.1, from which components related to motion and physiological noise were removed. This includes, in particular, removing components that showed significant correlation with the recorded breathing and cardiac patterns. Temporal filtering was performed using a low-pass filter with a cut-off frequency of 0.03 Hz. No normalization was performed during preprocessing, and all subsequent fMRI analysis was performed in the subject space. The MPRAGE structural images were co-registered to the fMRI images for each participant. The Automated Anatomical Labeling (AAL) atlas (Rolls et al., 2020) was used to identify primary and secondary olfactory regions in the brain according to the literature (Poellinger et al., 2001). The inverse deformation field was obtained to transform the regions-of-interest (ROI) identified in the AAL atlas from the MNI (Montreal Imaging Institute) space to the subject space. Since the OB is not included in the AAL atlas, it was manually delineated on the MPRAGE images for each participant. The manual segmentation of the OB was performed on all subjects by two researchers (XM and AGP) independently, who have been trained by senior neuroradiologists and have been performing OB segmentation in various studies in the group for over 3 years. After segmentation was completed, discrepancies between the two researchers were assessed and final measurements agreed upon. Figure 2 shows the 15 ROIs investigated in the current study overlaid on T2prep BOLD fMRI images from one subject. Note that only six coronal slices were provided in Figure 2 to illustrate the locations of the ROIs, but many ROIs can cover more slices. Most olfactory regions are best viewed in the coronal plane. To compare the quality of fMRI images in the OB, the OB was also identified on individual T2prep BOLD and GRE EPI images. To do that, the OB ROI from MPRAGE was overlaid on each fMRI image. A threshold of two standard deviations below the average signal intensity of the entire slice (not just the OB) was used, and voxels with intensities above this threshold within the MPRAGE OB ROI were counted in each fMRI image. Note that only the ROIs from MPRAGE were used for subsequent functional analysis.

Functional activation during olfactory stimulation in the whole brain was detected using the non-parametric Kolmogorov–Smirnov (KS) two-sample test (Siegel and Castellan, 1988) (adjusted *P* < 0.01). Compared to the commonly used general linear model (GLM), previous olfactory fMRI studies (Sobel et al., 2000; Poellinger et al., 2001) have shown that the KS statistic may be more suitable for detecting functional activations with strong habituation effects. Note that the KS statistic is also suitable for detecting functional activations without habituation effects (Sobel et al., 2000; Poellinger et al., 2001). Nuisance parameters such as motion and breathing and cardiac pattern that are usually controlled in GLM have been removed using the ICA based denoising approach (Salimi-Khorshidi et al., 2014) described above. Relative signal changes (ΔS/S) between the stimulus-on and stimulus-off periods were calculated for each voxel. Note that due to the transition period following the cessation of stimulus that usually lasts for 50–100% of the stimulus-on period (Hua et al., 2011; van Zijl et al., 2012), only signals from the second half of the stimulus-off period were included when calculating ΔS/S. Because the two-sample KS statistic detects both positive and negative activation during stimulation (Georgiopoulos et al., 2018), the activated voxels were separated into positively (ΔS/S > 0) and negatively (ΔS/S < 0) activated voxels subsequently. Temporal signal-to-noise ratio (tSNR) was calculated as the signal divided by standard deviation along the time course in each voxel. CNR was defined as the product of tSNR and ΔS/S from the 1st block. The CNR comparison was conducted in voxels that were positively activated in either GRE EPI or T2prep fMRI scans in each participant. We chose to use the combined activated voxels from both scans because in some ROIs with significant susceptibility artifacts such as the OB, GRE EPI scans showed little activation. The same voxels were used in both fMRI methods and results in each ROI are shown.

### Statistical Analysis

The comparison between GRE EPI and T2prep BOLD fMRI was performed in a subset of participants (*n* = 5). Power analysis was performed using the approach described by Cohen et al. (Cohen, 1988) based on the average effect size (approximately 1.3) reported in previous studies (Hua et al., 2017; Miao et al., 2020) to ensure that this sample size can provide a power of 0.8 with significance set at *a* = 0.05 (type I error, two tailed) for the CNR comparison between these two methods. This is consistent with similar technical studies using the same MRI methods performed at 3T (Hua et al., 2017; Miao et al., 2020). As the CNR difference between the two fMRI methods in the OB is mainly caused by the well-known susceptibility artifacts from the nearby nasal cavity (Yang et al., 1997; Sobel et al., 1998, 2000; Poellinger et al., 2001; Wang et al., 2010; Zong et al., 2014; Lu et al., 2018), the effect sizes in the current study on 7T are expected to be greater than previous 3T studies (Hua et al., 2017; Miao et al., 2020). We therefore believe that this sample size is sufficient for this technical comparison.

Paired *t*-tests were performed to compare CNR from GRE EPI BOLD and T2prep BOLD fMRI. Effect size was estimated with Cohen’s d. One-way repeated-measures analysis of variance (ANOVA) was conducted to examine differences of ΔS/S among the three blocks of the olfactory paradigm. All statistical tests were corrected for multiple comparisons by controlling the false-discovery rate (adjusted *P* < 0.05).

### Reproducibility

In all participants (*n* = 14), the same T2prep BOLD fMRI scans and analysis were repeated using the same functional paradigm once to assess its reproducibility. The second T2prep BOLD scan (re-scan) was performed on the same scanner in 3–6 weeks after the first scan for each participant. Dice coefficients between the maps of activated voxels from the scan and re-scan of the same subjects were calculated to evaluate the reproducibility of spatial locations of the activated clusters (Sair et al., 2016). The value of a Dice coefficient ranges from 0, indicating no spatial overlap between the scan and re-scan results, to 1, indicating complete overlap. Intraclass correlation coefficient (ICC) was calculated to evaluate the reproducibility of ΔS/S between the scan and re-scan results of T2prep BOLD fMRI in the same subjects. In each ROI, activated voxels (positively or negatively) from the first scan were overlaid on the second scan from the same subject. The ICC of ΔS/S from these same voxels were calculated for each ROI and each subject. The definition of ICC in a textbook (Rosner, 2011) was adopted. The procedure used here is identical to that in our previous reproducibility studies (Landman et al., 2011).

## Results

### Comparison of GRE EPI BOLD and T2prep BOLD fMRI

**FIGURE 3:**
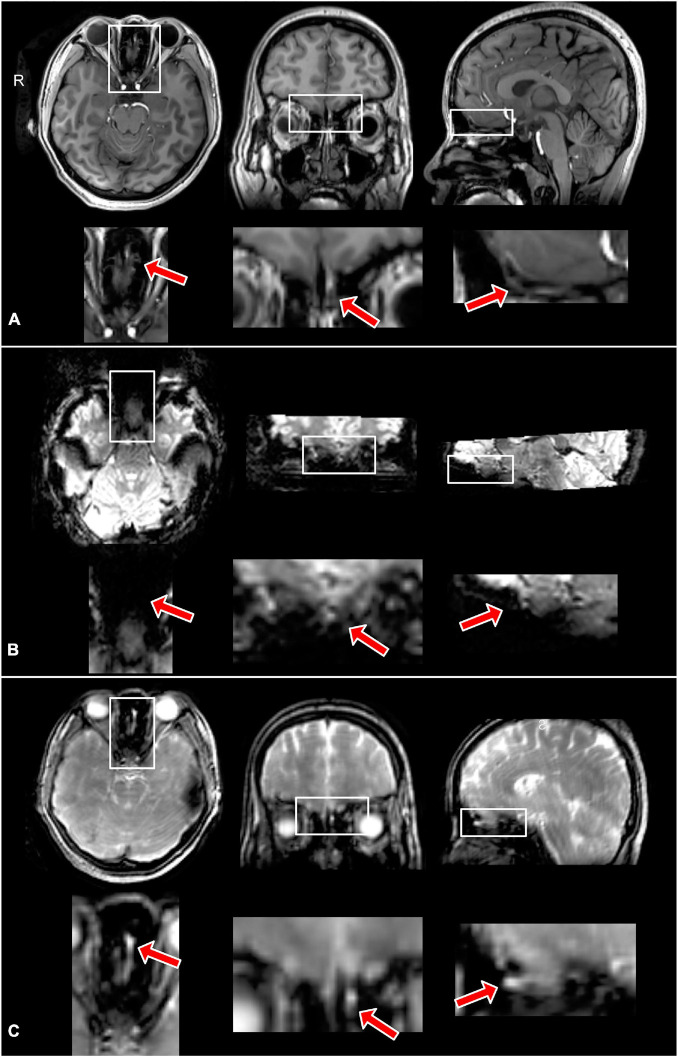
Typical MPRAGE **(A)**, GRE EPI BOLD **(B)**, and T2prep BOLD **(C)** images from one subject. As the size of the olfactory bulb is small, the regions in the white boxes covering the areas around the olfactory bulb are magnified in the panel below each image for better visualization. The red arrows indicate the location of the olfactory bulb. The olfactory bulb can be clearly identified in the MPRAGE and T2prep images, but not in the GRE EPI images due to substantial susceptibility artifacts. All images are from the same location. Note that the eye ball region in the GRE EPI **(B)** images also showed high distortion and dropout [the outline of the eye balls can be seen at the approximately same location as images shown in panels **(A,C)**].

**FIGURE 4:**
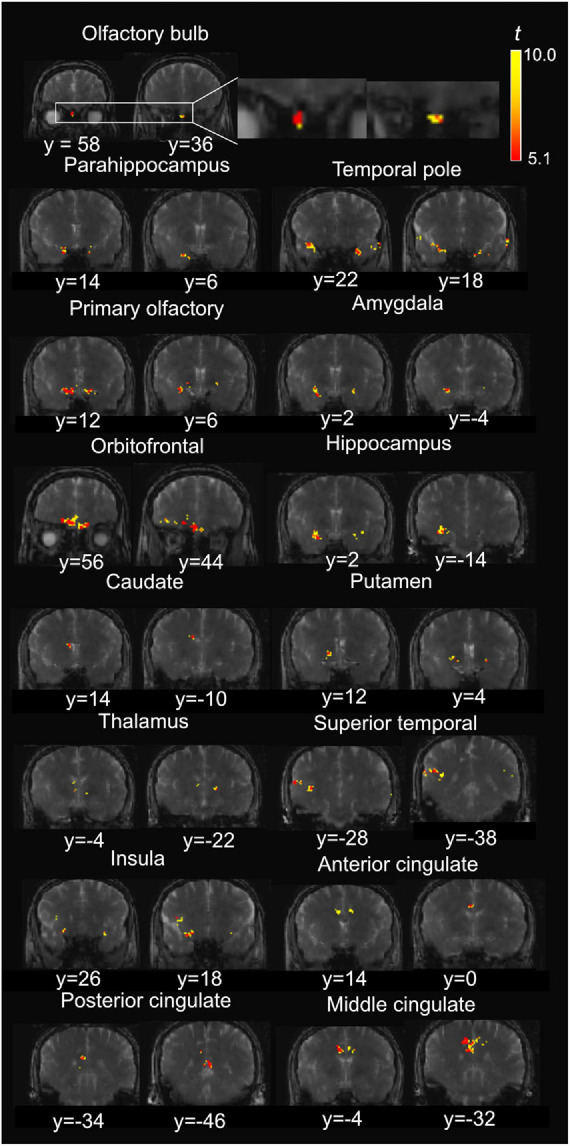
Representative positive activation maps from T2prep BOLD fMRI scans during the olfactory stimulation from one participant. The *t*-scores of significantly positively activated voxels are overlaid on the T2prep BOLD images from the same participant. Two slices were shown for each ROI. But the entire activated cluster in each ROI covered more slices.

Figure 3 shows the typical image quality of GRE EPI BOLD and T2prep BOLD fMRI from one subject. The T1-weighted MPRAGE images serve as an anatomical reference with minimal distortion and dropout. In the GRE EPI BOLD images, the susceptibility artifacts in the OB were substantial, showing signal dropout and geometric distortion caused by the nearby cavities. These artifacts were significantly reduced in T2prep BOLD images from the same subject. On average, the OB can be clearly depicted on 4 ± 2 (*n* = 5) slices of T2prep images, and the group-averaged size of the OB was 23 ± 5 (*n* = 5) voxels or 77 ± 16 mm^3^ (*n* = 5) on T2prep images, consistent with literature values for healthy human subjects (Herzallah et al., 2013; Alarabawy et al., 2016). Similar results were obtained on MPRAGE images (4 ± 2 slices and 22 ± 7 voxels, *n* = 5). On GRE EPI images, the OB can only be identified on 1 ± 1 (*n* = 5) slices and 5 ± 3 (*n* = 5) voxels, significantly less than T2prep and MPRAGE (*P* < 0.01). Table 1 shows the quantitative CNR results. T2prep showed significantly greater CNR than GRE EPI in the OB, orbitofrontal cortex and the temporal pole. In regions that are less affected by susceptibility effects, GRE EPI showed similar or better CNR than T2prep. In middle cingulate, GRE EPI showed significantly greater CNR than T2prep. The number of voxels in the OB reported in Table 1 is small as it is a very small region. Typical activation maps from T2prep BOLD and GRE EPI BOLD fMRI scans are shown in Figure 4 and Supplementary Figure S2, respectively.

**TABLE 1:** Comparison of contrast-to-noise ratio (CNR) between T2prep BOLD and GRE EPI BOLD fMRI (*n* = 5).

ROI	ROI size (# voxel)	T2prep BOLD	GRE EPI BOLD	*P*	Relative difference (%)	Effect size
Olfactory bulb	15.4 ± 5.1	1.3 ± 0.3	0.5 ± 0.1	0.02*	183.3	4.7
Parahippocampus	268.1 ± 83.4	0.9 ± 0.2	0.8 ± 0.1	0.57	8.5	0.4
Temporal pole	785.0 ± 245.3	1.7 ± 0.3	0.4 ± 0.1	0.01*	296.5	5.3
Primary olfactory	67.0 ± 21.2	1.1 ± 0.3	1.1 ± 0.2	0.68	6.1	0.3
Amygdala	57.0 ± 16.0	0.5 ± 0.1	1.7 ± 0.7	0.15	−68.0	−2.4
Orbitofrontal	263.0 ± 93.0	0.9 ± 0.1	0.2 ± 0.1	<0.01*	417.7	7.5
Hippocampus	212.1 ± 71.7	0.9 ± 0.2	2.1 ± 0.5	0.16	−58.4	−3.4
Caudate	185.0 ± 58.5	1.1 ± 0.2	1.6 ± 0.4	0.44	−27.0	−1.4
Putamen	259.1 ± 91.4	0.7 ± 0.1	0.7 ± 0.2	0.62	6.9	0.3
Thalamus	244.9 ± 73.9	0.9 ± 0.2	1.5 ± 0.3	0.23	−39.4	−2.0
Superior temporal	498.8 ± 163.8	0.7 ± 0.2	0.3 ± 0.1	0.07	125.4	3.2
Insula	458.2 ± 149.4	1.0 ± 0.2	0.9 ± 0.1	0.71	6.8	0.4
Anterior cingulate	283.2 ± 95.0	0.9 ± 0.2	1.2 ± 0.2	0.24	−24.6	−1.4
Posterior cingulate	289.5 ± 97.0	0.7 ± 0.1	1.6 ± 0.4	0.12	−58.7	−3.0
Middle cingulate	413.3 ± 130.5	0.9 ± 0.1	2.3 ± 0.4	0.02*	−62.3	−4.9

Mean ± standard error. *P < 0.05.

*The same voxels were used in both fMRI methods. The number of voxels in the olfactory bulb is small as it is a very small region.*

*Relative difference was defined as 100 × (mean CNR in T2prep – mean CNR in EPI)/(mean CNR in EPI) %.*

*Effect size was calculated using Cohen’s *d* = (mean CNR in T2prep – mean CNR in EPI)/s, where s is the pooled standard deviation of the two groups.*

### Olfactory fMRI Results Using T2prep BOLD fMRI

**FIGURE 5:**
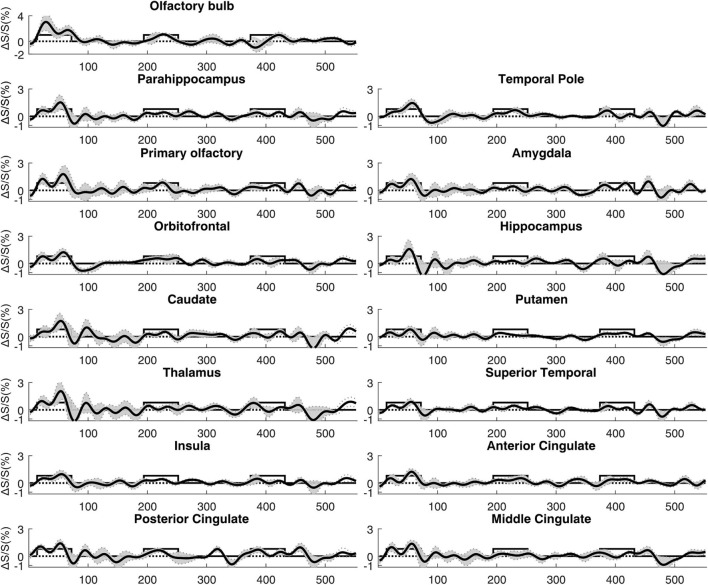
Average time courses of fMRI signal changes from all participants (*n* = 14). For each participant, the time course was averaged over positively activated voxels in each ROI. The gray shaded area indicates the inter-subject standard error. The boxcar curve illustrates the timing of the olfactory paradigm described in Figure 1. The order of the ROIs was arranged to be identical to Figure 4.

Figure 4 shows a representative activation map from one participant. Positively activated voxels in the OB and associated olfactory regions were overlaid on the T2prep BOLD images. The average number of positively activated voxels in the OB was 15 ± 5 (*n* = 14) on T2prep images. The group averaged time courses of relative signal changes (ΔS/S) during the paradigm from positively activated voxels in each ROI are displayed in Figure 5. Table 2 summarizes the quantitative results (ΔS/S) from positively activated voxels in each ROI. As the same olfactory stimulation was repeated for three blocks (Figure 1), ΔS/S from each block was calculated and compared. In all ROIs except for the putamen, ΔS/S decreased significantly (*P* < 0.05) during the 2nd and 3rd blocks compared to that during the 1st block. In putamen, ΔS/S showed a similar decreasing trend but failed to reach significance (*P* = 0.06). The magnitude of ΔS/S in the OB during the 1st block was greater than that in the other regions. Within the 1st block, the time course of ΔS/S in the OB showed an initial increase during the first half of the block (∼30s) followed by a substantially smaller ΔS/S during the second half of the block. In all the other ROIs, ΔS/S within the 1st block showed an initial increase during the first half of the block (∼30s) followed by a second peak with similar magnitude during the second half of the block. The rising and decaying times for both peaks from the time courses were similar among all regions. Supplementary Figure S4 shows the map of positively activated voxels combined from all participants (*n* = 14) after individual maps were normalized to the MNI space.

**TABLE 2:** Quantitative T2prep BOLD fMRI results from positively activated voxels from all subjects (*n* = 14).

ROI	Activated voxels (#)	ΔS/S (%)*			P**	Relative difference (%)***	
		1st block	2nd block	3rd block		2nd vs. 1st	3rd vs. 1st
Olfactory bulb	14.8 ± 5.3	2.2 ± 0.7	0.8 ± 0.5	−0.2 ± 0.5	0.02	−65 ± 56	−110 ± 45
Parahippocampus	244.1 ± 87.8	0.8 ± 0.3	0.2 ± 0.1	0.2 ± 0.1	0.02	−75 ± 42	−80 ± 45
Temporal Pole	766.0 ± 267.3	0.8 ± 0.3	0.4 ± 0.1	0.2 ± 0.1	0.03	−43 ± 31	−80 ± 40
Primary olfactory	65.0 ± 23.3	1.3 ± 0.6	0.2 ± 0.1	0.2 ± 0.1	0.02	−78 ± 54	−81 ± 52
Amygdala	54.0 ± 19.8	0.7 ± 0.3	0.3 ± 0.1	0.2 ± 0.1	0.05	−62 ± 66	−67 ± 59
Orbitofrontal	248.0 ± 83.1	0.8 ± 0.3	0.5 ± 0.1	0.2 ± 0.1	0.02	−39 ± 38	−68 ± 32
Hippocampus	203.7 ± 70.3	0.7 ± 0.2	0.2 ± 0.1	0.1 ± 0.1	0.02	−71 ± 42	−81 ± 48
Caudate	181.0 ± 57.4	1.1 ± 0.4	0.2 ± 0.1	0.2 ± 0.2	0.01	−79 ± 35	−79 ± 22
Putamen	237.7 ± 80.7	0.4 ± 0.3	0.3 ± 0.2	0.2 ± 0.1	0.06	−23 ± 69	−48 ± 62
Thalamus	220.4 ± 80.8	1.2 ± 0.4	0.1 ± 0.1	0.1 ± 0.1	0.01	−87 ± 41	−88 ± 41
Superior temporal	482.0 ± 177.2	0.4 ± 0.1	0.3 ± 0.1	0.1 ± 0.1	0.02	−35 ± 32	−79 ± 36
Insula	432.0 ± 145.4	0.6 ± 0.2	0.2 ± 0.1	0.1 ± 0.1	0.03	−66 ± 47	−75 ± 41
Anterior cingulate	269.2 ± 95.2	0.8 ± 0.2	0.3 ± 0.1	0.1 ± 0.1	0.02	−61 ± 31	−80 ± 44
Posterior cingulate	284.0 ± 94.4	0.9 ± 0.3	0.4 ± 0.1	0.0 ± 0.1	0.02	−50 ± 27	−90 ± 29
Middle cingulate	383.3 ± 144.0	0.8 ± 0.3	0.2 ± 0.1	0.1 ± 0.1	0.02	−71 ± 30	−88 ± 42

*Mean ± standard error.*

*ΔS/S averaged over the 1st, 2nd, and 3rd blocks during the olfactory paradigm shown in Figure 1 was calculated separately.

**P values from one-way repeated-measures analysis of variance (ANOVA) to examine differences of ΔS/S among the three blocks of the olfactory paradigm.

***Relative difference = 100 × (ΔS/S 2nd or 3rd block – ΔS/S 1st block)/group mean of ΔS/S 1st block %.

*The number of voxels in the olfactory bulb is small as it is a very small region.*

**FIGURE 6:**
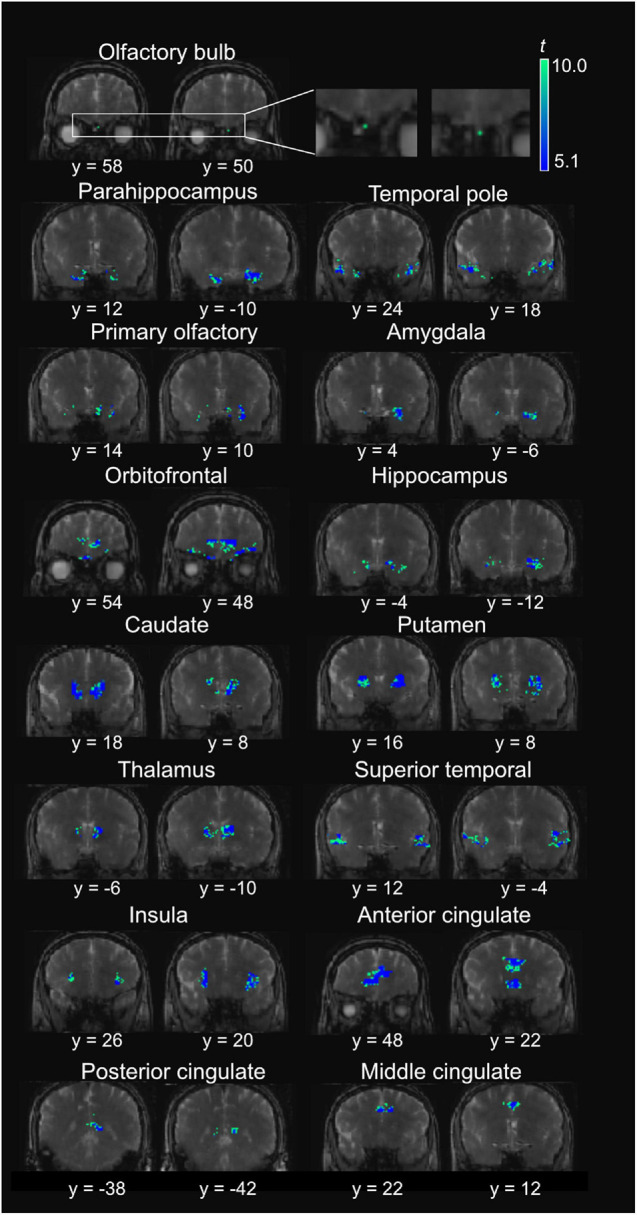
Representative negative activation maps from T2prep BOLD fMRI scans during the olfactory stimulation from one participant. The *t*-scores of significantly negatively activated voxels are overlaid on the T2prep BOLD images from the same participant. Two slices are shown for each ROI. But the entire activated cluster in each ROI covered more slices.

**FIGURE 7:**
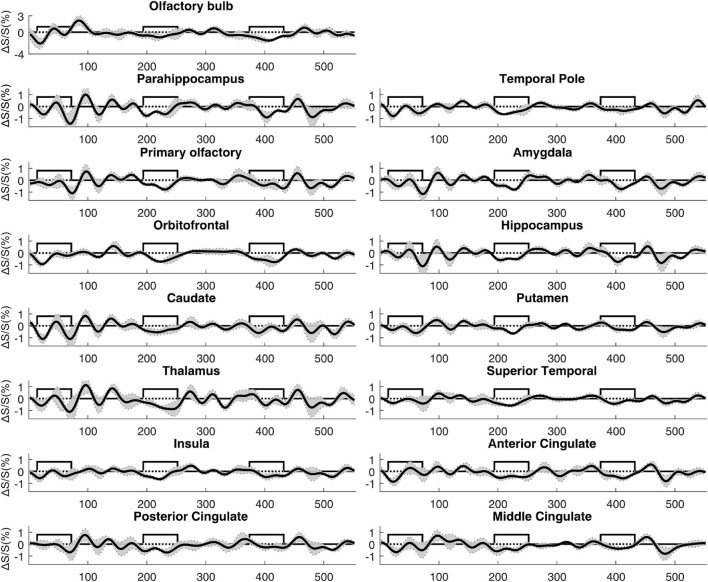
Average time courses of fMRI signal changes from all participants (*n* = 14). For each participant, the time course was averaged over negatively activated voxels in each ROI. The gray shaded area indicates the inter-subject standard error. The boxcar curve illustrates the timing of the olfactory paradigm described in Figure 1. The order of the ROIs was arranged to be identical to Figure 6.

Negatively activated voxels were also observed in all ROIs shown in Figure 2. By definition in section “Materials and Methods,” negatively and positively activated voxels are two mutually exclusive subsets of voxels in each ROI. Figure 6 shows a representative activation map from one participant. Negatively activated voxels in the OB and associated olfactory regions were overlaid on the T2prep BOLD images. The average number of negatively activated voxels in the OB was 8 ± 5 (*n* = 14). The group averaged time courses of ΔS/S during the paradigm from negatively activated voxels in each ROI are displayed in Figure 7. Table 3 summarizes the quantitative results (ΔS/S) from negatively activated voxels in each ROI. Compared to positively activated voxels, in all ROIs, the absolute values of ΔS/S during the 1st block were smaller for negatively activated voxels. ΔS/S during the 1st, 2nd, and 3rd blocks were not substantially different for negatively activated voxels. Within the 1st block, time courses of ΔS/S averaged from the negatively activated voxels in most ROIs showed a bi-phasic pattern similar to that from positively activated voxels. Supplementary Figure S5 shows the map of negatively activated voxels combined from all participants (*n* = 14) after individual maps were normalized to the MNI space.

**TABLE 3:** Quantitative T2prep BOLD fMRI results from negatively activated voxels from all subjects (*n* = 14).

ROI	Activated voxels (#)	ΔS/S (%)*			P**	Relative difference (%)***	
		1st block	2nd block	3rd block		2nd vs. 1st	3rd vs. 1st
Olfactory bulb	8.1 ± 5.5	−0.8 ± 0.6	−0.5 ± 0.4	−0.9 ± 0.3	0.45	−38 ± 122	38 ± 62
Parahippocampus	134.6 ± 38.1	−0.4 ± 0.2	−0.5 ± 0.3	−0.3 ± 0.2	0.53	6 ± 82	−42 ± 42
Temporal pole	444.6 ± 153.9	−0.6 ± 0.3	−0.4 ± 0.3	−0.2 ± 0.2	0.29	−−32 ± 55	−68 ± 31
Primary olfactory	39.7 ± 12.9	−0.5 ± 0.1	-0.5 ± 0.2	−0.2 ± 0.1	0.25	20 ± 45	−46 ± 35
Amygdala	34.3 ± 11.1	−0.5 ± 0.2	−0.5 ± 0.1	−0.3 ± 0.1	0.33	12 ± 62	−33 ± 42
Orbitofrontal	137.6 ± 51.8	−0.6 ± 0.1	−0.5 ± 0.2	−0.4 ± 0.1	0.58	−3 ± 52	−8 ± 38
Hippocampus	95.3 ± 32.3	−0.3 ± 0.1	−0.4 ± 0.2	−0.3 ± 0.1	0.46	102 ± 111	30 ± 66
Caudate	92.2 ± 31.0	−0.6 ± 0.3	−0.5 ± 0.2	−0.3 ± 0.2	0.29	−25 ± 51	−56 ± 41
Putamen	116.8 ± 44.7	−0.4 ± 0.1	−0.3 ± 0.1	−0.1 ± 0.1	0.06	−42 ± 36	−82 ± 22
Thalamus	149.7 ± 45.4	−0.6 ± 0.2	−0.6 ± 0.1	−0.2 ± 0.2	0.16	30 ± 50	−68 ± 42
Superior temporal	248.8 ± 87.8	−0.3 ± 0.1	−0.3 ± 0.2	−0.1 ± 0.1	0.55	52 ± 102	−40 ± 81
Insula	255.4 ± 91.3	−0.4 ± 0.2	−0.4 ± 0.2	−0.2 ± 0.1	0.46	25 ± 87	−52 ± 68
Anterior cingulate	145.2 ± 46.7	−0.4 ± 0.2	−0.5 ± 0.2	−0.3 ± 0.2	0.50	51 ± 79	5 ± 99
Posterior cingulate	170.1 ± 57.3	−0.3 ± 0.2	−0.5 ± 0.3	−0.2 ± 0.2	0.56	152 ± 189	6 ± 115
Middle cingulate	194.7 ± 56.7	−0.4 ± 0.2	−0.4 ± 0.2	−0.3 ± 0.2	0.77	−20 ± 121	−41 ± 92

*Mean ± standard error.*

*ΔS/S averaged over the 1^*st*^, 2^*nd*^, and 3^*rd*^ blocks during the olfactory paradigm shown in Figure 1 was calculated separately.

**P values from one-way repeated-measures analysis of variance (ANOVA) to examine differences of ΔS/S among the three blocks of the olfactory paradigm.

***Relative difference = 100 × (ΔS/S 2nd or 3rd block – ΔS/S 1st block)/group mean of ΔS/S 1st block %.

*The number of voxels in the olfactory bulb is small as it is a very small region.*

Tables 4, 5 summarize the reproducibility results for T2prep BOLD fMRI. The spatial locations of the positively and negatively activated clusters in all ROIs showed a good reproducibility with Dice coefficients ranging from 0.87 to 0.93. The relative signal changes (ΔS/S) in all ROIs and all blocks showed comparable reproducibility (ICC) to BOLD fMRI measures assessed in our previous 3T study (Landman et al., 2011).

**TABLE 4:** Reproducibility for positively activated voxels in T2prep BOLD fMRI (*n* = 14).

ROI	Dice* coefficient	ICC of ΔS/S**		
		1st block	2nd block	3rd block
Olfactory bulb	0.91 ± 0.06	0.90 ± 0.06	0.89 ± 0.05	0.88 ± 0.06
Parahippocampus	0.89 ± 0.08	0.83 ± 0.07	0.85 ± 0.06	0.83 ± 0.06
Temporal pole	0.93 ± 0.04	0.86 ± 0.07	0.86 ± 0.08	0.86 ± 0.08
Primary olfactory	0.93 ± 0.04	0.88 ± 0.05	0.89 ± 0.08	0.87 ± 0.08
Amygdala	0.87 ± 0.09	0.82 ± 0.08	0.88 ± 0.09	0.86 ± 0.07
Orbitofrontal	0.91 ± 0.06	0.89 ± 0.06	0.89 ± 0.06	0.88 ± 0.07
Hippocampus	0.93 ± 0.05	0.88 ± 0.06	0.89 ± 0.05	0.88 ± 0.05
Caudate	0.92 ± 0.07	0.86 ± 0.07	0.87 ± 0.08	0.88 ± 0.06
Putamen	0.89 ± 0.07	0.85 ± 0.08	0.82 ± 0.09	0.83 ± 0.09
Thalamus	0.88 ± 0.07	0.84 ± 0.06	0.84 ± 0.06	0.85 ± 0.08
Superior temporal	0.92 ± 0.06	0.85 ± 0.06	0.86 ± 0.06	0.88 ± 0.05
Insula	0.93 ± 0.06	0.88 ± 0.04	0.88 ± 0.05	0.87 ± 0.07
Anterior cingulate	0.87 ± 0.09	0.83 ± 0.07	0.83 ± 0.07	0.86 ± 0.08
Posterior cingulate	0.87 ± 0.09	0.83 ± 0.08	0.85 ± 0.08	0.85 ± 0.06
Middle cingulate	0.88 ± 0.10	0.83 ± 0.07	0.83 ± 0.07	0.86 ± 0.06

*Mean ± standard error.*

*Dice coefficients between the maps of positively activated voxels from the scan and re-scan of the same subjects were calculated to evaluate the reproducibility of spatial locations of the activated clusters. The value of a Dice coefficient ranges from 0, indicating no spatial overlap between the scan and re-scan results, to 1, indicating complete overlap.

**Intraclass correlation coefficient (ICC) of ΔS/S between the scan and re-scan results of T2prep BOLD fMRI in the same subjects.

**TABLE 5:** Reproducibility for negatively activated voxels in T2prep BOLD fMRI (*n* = 14).

ROI	Dice* coefficient	ICC of ΔS/S**		
		1st block	2nd block	3rd block
Olfactory bulb	0.91 ± 0.06	0.91 ± 0.06	0.90 ± 0.06	0.90 ± 0.04
Parahippocampus	0.89 ± 0.07	0.86 ± 0.06	0.86 ± 0.07	0.85 ± 0.06
Temporal pole	0.92 ± 0.06	0.90 ± 0.07	0.88 ± 0.07	0.89 ± 0.07
Primary olfactory	0.92 ± 0.06	0.90 ± 0.06	0.90 ± 0.07	0.91 ± 0.06
Amygdala	0.87 ± 0.08	0.87 ± 0.06	0.86 ± 0.07	0.89 ± 0.06
Orbitofrontal	0.91 ± 0.07	0.89 ± 0.05	0.90 ± 0.05	0.89 ± 0.08
Hippocampus	0.92 ± 0.06	0.88 ± 0.05	0.88 ± 0.05	0.88 ± 0.06
Caudate	0.93 ± 0.06	0.89 ± 0.04	0.90 ± 0.05	0.87 ± 0.06
Putamen	0.88 ± 0.07	0.86 ± 0.06	0.84 ± 0.07	0.84 ± 0.07
Thalamus	0.88 ± 0.06	0.86 ± 0.07	0.86 ± 0.06	0.88 ± 0.08
Superior temporal	0.91 ± 0.06	0.89 ± 0.05	0.89 ± 0.06	0.89 ± 0.08
Insula	0.92 ± 0.05	0.88 ± 0.05	0.88 ± 0.05	0.87 ± 0.05
Anterior cingulate	0.87 ± 0.09	0.85 ± 0.07	0.85 ± 0.07	0.85 ± 0.07
Posterior cingulate	0.87 ± 0.08	0.85 ± 0.07	0.85 ± 0.05	0.88 ± 0.05
Middle cingulate	0.88 ± 0.09	0.87 ± 0.03	0.85 ± 0.08	0.85 ± 0.08

*Mean ± standard error.*

*Dice coefficients between the maps of negatively activated voxels from the scan and re-scan of the same subjects were calculated to evaluate the reproducibility of spatial locations of the activated clusters. The value of a Dice coefficient ranges from 0, indicating no spatial overlap between the scan and re-scan results, to 1, indicating complete overlap.

**Intraclass correlation coefficient (ICC) of ΔS/S between the scan and re-scan results of T2prep BOLD fMRI in the same subjects.

## Discussion

We demonstrated that the whole brain T2prep BOLD fMRI technique can detect functional activations in response to olfactory stimulation in primary and secondary olfactory regions in healthy human subjects at 7T. Neuronal activation triggered cerebral blood flow (CBF) increase during odor stimulation has been demonstrated in the rodent OB using two-photon imaging and electrophysiology recordings (Chaigneau et al., 2007). Although functional activities in the OB have been measured in various animal studies (Xu et al., 2000, 2003, 2005; Boyett-Anderson et al., 2003; Cross et al., 2004; Schafer et al., 2005; Martin et al., 2007; Jia et al., 2014; Li et al., 2014; Poplawsky and Kim, 2014; Berns et al., 2015; Zhao et al., 2015, 2016, 2017; Murphy et al., 2016; Muir et al., 2019), to our knowledge, this is the first fMRI study to detect functional activation in the OB in human subjects using whole brain BOLD fMRI. Previous reports that detected functional activation in the OB are either animal studies (Xu et al., 2000, 2003, 2005; Boyett-Anderson et al., 2003; Cross et al., 2004; Schafer et al., 2005; Martin et al., 2007; Jia et al., 2014; Li et al., 2014; Poplawsky and Kim, 2014; Berns et al., 2015; Zhao et al., 2015, 2016, 2017; Murphy et al., 2016; Muir et al., 2019) or human scans with partial brain coverage (Fournel et al., 2020). T2prep BOLD was originally developed at 7T (Hua et al., 2014) to minimize susceptibility artifacts commonly seen in EPI based fMRI methods that are more prominent at higher magnetic fields, and was later on applied at 3T for fMRI in individuals with metallic head implants (Miao et al., 2020) and in patients undergoing presurgical brain mapping (Hua et al., 2017). In brain regions that are less affected by susceptibility artifacts such as the visual and motor cortices, GRE EPI BOLD still has better sensitivity. However, when susceptibility artifacts become prominent in some frontal and temporal areas and regions near metal objects, the sensitivity of GRE EPI BOLD drops substantially, whereas the BOLD sensitivity is largely preserved in the entire brain in T2prep BOLD (Hua et al., 2014, 2017; Miao et al., 2020). Our data in this study showed that several olfactory regions that are significantly affected by susceptibility artifacts in EPI due to nearby air cavities and bone structures had significantly enhanced CNR in T2prep BOLD compared to GRE EPI. These regions include the OB, orbitofrontal cortex and temporal cortex. In other regions less affected by susceptibility artifacts, CNR values were similar for both methods, or higher in GRE EPI. Although the current study was performed at 7T, the same methodology has been implemented on 3T human MRI systems as well (Hua et al., 2017; Miao et al., 2020). In addition to healthy subjects, we have also been using the same methodology on 3T and 7T in clinical populations with olfactory deficits such as individuals with Parkinson’s disease, Alzheimer’s disease, and schizophrenia.

Habituation to olfactory stimuli is a well-known phenomenon (Pellegrino et al., 2017) in which an attenuation of responses to prolonged and/or repeated olfactory stimulation is observed. In this study, a relatively long (60 s) olfactory stimulation was repeated three times in each participant in order to evaluate habituation effects in different brain regions. To detect functional activation with anticipated habituation during the period of stimulation, the non-parametric KS two-sample test was adopted. Strong habituation effects may lead to greater signal variance during the stimulus-on periods than the stimulus-off periods. The KS statistic is considered to be highly sensitive to this difference in signal variance (Zhao et al., 1997), and therefore is more suitable for olfactory fMRI analysis than the commonly used GLM approach in most task based fMRI analysis, as demonstrated in previous human olfactory fMRI studies (Sobel et al., 2000; Poellinger et al., 2001). In our data from positively activated voxels, all regions investigated showed reduced responses in the 2nd and 3rd blocks compared to the 1st block. This is consistent with previous studies in humans and animals using imaging (Wilson, 1998; Yang X. et al., 1998; Sobel et al., 2000; Xu et al., 2000, 2003, 2005; Poellinger et al., 2001; Kida et al., 2002; Boyett-Anderson et al., 2003; Cross et al., 2004; Schafer et al., 2005, 2006; Martin et al., 2007; Jia et al., 2014; Li et al., 2014; Poplawsky and Kim, 2014; Berns et al., 2015; Zhao et al., 2015, 2016, 2017; Murphy et al., 2016; Muir et al., 2019) and electrophysiology recordings (Wilson, 1998; Chaudhury et al., 2010). Within the 1st block, all regions showed a bi-phasic pattern with two distinct peaks during the first and second halves of the 1st block. Comparing to the first peak, the second peak was much weaker in the OB than the other regions. The bi-phasic pattern has been reported in previous olfactory fMRI studies in rats (Zhao et al., 2016), where it was more prominent in higher olfactory regions such as the piriform cortex than in the OB, and was hypothesized to represent a post-inhibition excitation rebound (Zhao et al., 2016). Also, whether such bi-phasic pattern is affected by physiological noise sources warrant further investigation. In our data, the magnitude of the first peak was greater in the OB than in the other regions, but the time courses showed similar shapes among all regions. Overall, we did not observe a significant difference in habituation effects between the OB and the other olfactory regions in the current human study, whereas many previous animal studies have shown less pronounced habituation effects in the OB compared to higher olfactory regions (Wilson, 1998; Yang Q.X. et al., 1998; Xu et al., 2000, 2005; Kida et al., 2002; Schafer et al., 2005, 2006; Chaudhury et al., 2010; Zhao et al., 2015, 2016, 2017). One possible reason may be the pulsed pattern during the stimulus-on period used in the olfactory stimulation paradigm in this study. The duration of a single pulse (1s) may be too short to differentiate the habituation effects in the OB and higher olfactory regions, whereas the hemodynamic responses from BOLD fMRI in response to consecutive pulses may overlap temporally due to a short inter-pulse interval (2s), which could mask any potential difference between the OB and higher olfactory regions. Other possible factors may include species differences (human in the current study, non-human primate, and rodent in previous studies), choice of odorant and its concentration, and the statistical approach used to identify activated voxels during olfactory stimulation (KS and conventional GLM). To our knowledge, this is the first study to characterize BOLD fMRI signals in the OB in human subjects. Additional studies are needed to investigate these factors and their influence on habituation effects in the OB and cortical olfactory regions.

In all regions investigated in this study, including the OB, a substantial subset of voxels showed decreased fMRI signals upon olfactory stimulation. The magnitude of the signal responses from negatively activated voxels were smaller than those from positively activated voxels in corresponding regions, which made the habituation effects less prominent in negatively activated voxels. Such negative activations were observed from our data using the same functional analysis pipeline as positive activations, and also showed a good intra-subject reproducibility. Nevertheless, the physiological origin of the negative activations is unclear. Previous studies using electrical recordings have shown that different neurons in the primary olfactory cortex can show either increasing activity, decreasing activity, or a combination of both in response to the same odor stimulation (Tanabe et al., 1975; Nemitz and Goldberg, 1983; Wilson, 1998), which may be one of the plausible explanations for our data. Such negative activation has also been observed in the piriform cortex of rats (Zhao et al., 2017), which may be explained by the characteristics of “sparse coding” and “global inhibition” in the olfactory processing of piriform cortex (Poo and Isaacson, 2009). Alternatively, a few other theories have been proposed for negative BOLD activations (Huber et al., 2014; Mullinger et al., 2014), which warrants further investigation in subsequent studies combining fMRI with additional electrophysiological recording and imaging techniques.

It is important to exclude potential false positive voxels in fMRI studies. In the current study, we adopted a well-established pre-processing pipeline for human fMRI and the KS method established in previous human olfactory fMRI studies (Sobel et al., 2000; Poellinger et al., 2001) for functional analysis. An ICA based denoising approach (Salimi-Khorshidi et al., 2014) was employed to remove major confounding factors such as motion, breathing and cardiac pattern and other physiological noise. The T2prep BOLD fMRI results showed a good intra-subject reproducibility comparable to previous human BOLD fMRI studies for both the spatial pattern and the temporal profile of signal changes detected during the olfactory paradigm. We feel that a comprehensive evaluation of the neuronal origin of the fMRI signals is beyond the scope of this technical work. However, we are currently conducting a subsequent study where the EBG technique (Iravani et al., 2020) will be used to provide some validation for the neuronal origin of the fMRI signal changes measured in the OB.

The goal of the current study is to evaluate the T2prep BOLD fMRI method as one of the alternative approaches for improving fMRI signals in olfactory regions affected by significant susceptible artifacts. Many other techniques are available to improve signals in high susceptible regions such as spin echo (SE) EPI, spiral MRI, and gradient spin echo (GRASE) MRI. Parallel imaging and multiband techniques can substantially shorten the echo train in EPI readout, and thus mitigate distortions to some extent. Many methods have been developed to reduce dropouts in GRE EPI (Frahm et al., 1988; Cho and Ro, 1992; Ordidge et al., 1994; Constable, 1995; Ojemann et al., 1997; Yang et al., 1997, 2006; Yang Q.X. et al., 1998; Constable and Spencer, 1999; Glover, 1999; Cordes et al., 2000; Devlin et al., 2000; Stenger et al., 2000; Merboldt et al., 2001; Deichmann et al., 2002, 2003; Gu et al., 2002; Wilson and Jezzard, 2003; Wilson et al., 2003; Heberlein and Hu, 2004; Robinson et al., 2004; Cusack et al., 2005; De Panfilis and Schwarzbauer, 2005; Juchem et al., 2006; Koch et al., 2006; Weiskopf et al., 2006, 2007; Du et al., 2007; Haines et al., 2010; Teeuwisse et al., 2012; Halai et al., 2014; Wastling and Barker, 2014) and distortion in EPI (Chang and Fitzpatrick, 1992; Weisskoff and Davis, 1992; Bowtell et al., 1994; Jezzard and Balaban, 1995; Morrell and Spielman, 1997; Robson et al., 1997; Wan et al., 1997; Reber et al., 1998; Chen and Wyrwicz, 1999, 2001; Jezzard and Clare, 1999; Kybic et al., 2000; Munger et al., 2000; Studholme et al., 2000; Andersson et al., 2001, 2003; Hutton et al., 2002; Ward et al., 2002; Zeng and Constable, 2002; Roopchansingh et al., 2003; Morgan et al., 2004; Zaitsev et al., 2004; Weiskopf et al., 2005; Gallichan et al., 2010; Holland et al., 2010; Chung et al., 2011; Oh et al., 2012; Visser et al., 2012; Ooi et al., 2013). It remains to be investigated in future studies which methods are most appropriate for specific applications.

The current study is designed to compare a new alternative method (3D T2prep BOLD) with the current method of choice for human fMRI (2D GRE EPI). Many aspects of the two methods differ from each other, which give rise to the different performance in each method. For instance, 3D and 2D acquisitions are intrinsically different. The spatial (voxel size) and temporal (TR) resolutions and the FOV were matched between the two methods for this comparison. Some other imaging parameters such as TE were optimized according to the BOLD theory for each method. The SENSE factor in 3D T2prep (SENSE = 3 × 3) was matched with 2D GRE EPI (SENSE = 3) in the AP direction, but was higher in T2prep in the FH direction, which should reduce tSNR in T2prep BOLD compared to GRE EPI. The approximate Ernst angle (70°) was used in GRE EPI. A lower flip angle was used in T2prep BOLD (4°), mainly due to the specific absorption rate (SAR) limitation, which should also reduce tSNR in T2prep BOLD compared to GRE EPI. The coverage of GRE EPI was partial brain as the multiband technique is currently not available on our 7T system. This is certainly not viewed as a disadvantage for GRE EPI. Once the multiband technique is implemented on our 7T, GRE EPI will be able to achieve the same spatial and temporal resolution as T2prep BOLD with whole brain coverage, although more acceleration will lead to lower tSNR compared to the current GRE EPI scan.

There are several limitations in this initial technical study using T2prep BOLD for olfactory fMRI in humans. First, sniff is a common confounding factor in olfactory fMRI. In our study, we ensured that every participant received and practiced instruction to breathe passively through the nose and avoid sniffing before the start of each experiment. In future studies, additional procedures, such as a target sniffing pattern, will be implemented to better control sniffing patterns during the experiments (Sobel et al., 2000). Secondly, the respiration pattern may vary individually which may confound the olfactory fMRI data. A standard respiratory belt provided by the vendor of our MRI system was used to record the respiration pattern for each participant during the fMRI experiments, which was later regressed out from fMRI data during analysis. In subsequent studies, one possible approach is to use a respiration-triggered olfactory fMRI technique (Wang et al., 2014) to provide a more precise estimate of the onsets of fMRI signals in response to olfactory stimulation. Thirdly, the olfactory stimulation paradigm can be re-designed to investigate how the various factors discussed above affect habituation in the OB and higher olfactory regions. Finally, the spatial resolution of 1.5 mm isotropic voxel in the current study is not sufficient to investigate layer dependent activities in the OB and other olfactory regions. Nevertheless, with the enhanced sensitivity from T2prep BOLD in the OB and other regions, fMRI with sub-millimeter spatial resolution focusing on the regions around the OB and primary olfactory cortex will be explored in subsequent studies.

## Conclusion

The OB and several other olfactory regions are difficult to image with conventional EPI based BOLD fMRI methods due to significant susceptibility artifacts. We demonstrated that T2prep BOLD fMRI can be an alternative method to reduce artifact and enhance functional sensitivity especially in the OB. The signal characteristics during olfactory stimulation detected using T2prep BOLD fMRI are generally consistent with literature and showed a good intra-subject reproducibility comparable to previous human BOLD fMRI studies. The methodology demonstrated in this technical study is expected to be useful for olfactory studies on brain regions that are often inaccessible with conventional fMRI approaches in healthy human subjects and patients with olfactory dysfunction in neurodegenerative and neuropsychiatric diseases.

## Data Availability Statement

The original contributions presented in the study are included in the article/Supplementary Material, further inquiries can be directed to the corresponding author.

## Ethics Statement

The studies involving human participants were reviewed and approved by the Johns Hopkins Institutional Review Board. The patients/participants provided their written informed consent to participate in this study.

## Author Contributions

XM and AGP: organization and execution of the study, statistical analysis, writing of the manuscript, review, and critique of the manuscript. SR, DC, AYP, and LR: organization and execution of the study, review, and critique of the manuscript. DL: writing of the manuscript, review, and critique of the manuscript. PZ, SB, and DY: conception and design, review, and critique of the manuscript. VK: conception and design, organization and execution of the study, review, and critique of the manuscript. JH: conception and design, organization and execution of the study, statistical analysis, writing of the manuscript, review, and critique of the manuscript. All authors contributed to the article and approved the submitted version.

## Conflict of Interest

Equipment used in the study was manufactured by Philips Healthcare and Whiff LLC. PZ is a paid lecturer for the Philips Healthcare and has technology licensed to them. This arrangement has been approved by Johns Hopkins University in accordance with its conflict of interest policies. The remaining authors declare that the research was conducted in the absence of any commercial or financial relationships that could be construed as a potential conflict of interest.

## Publisher’s Note

All claims expressed in this article are solely those of the authors and do not necessarily represent those of their affiliated organizations, or those of the publisher, the editors and the reviewers. Any product that may be evaluated in this article, or claim that may be made by its manufacturer, is not guaranteed or endorsed by the publisher.
