# Supplementary material for: Functional Activities Detected in the Olfactory Bulb and Associated Olfactory Regions in the Human Brain Using T2-Prepared BOLD Functional MRI at 7T
Source: Front Neurosci. 2021 Sep 13;15:723441. doi: 10.3389/fnins.2021.723441 (PMC8476065; doi:10.3389/fnins.2021.723441)
Supplement: Supplementary file 1 [file Data_Sheet_1.docx]

**
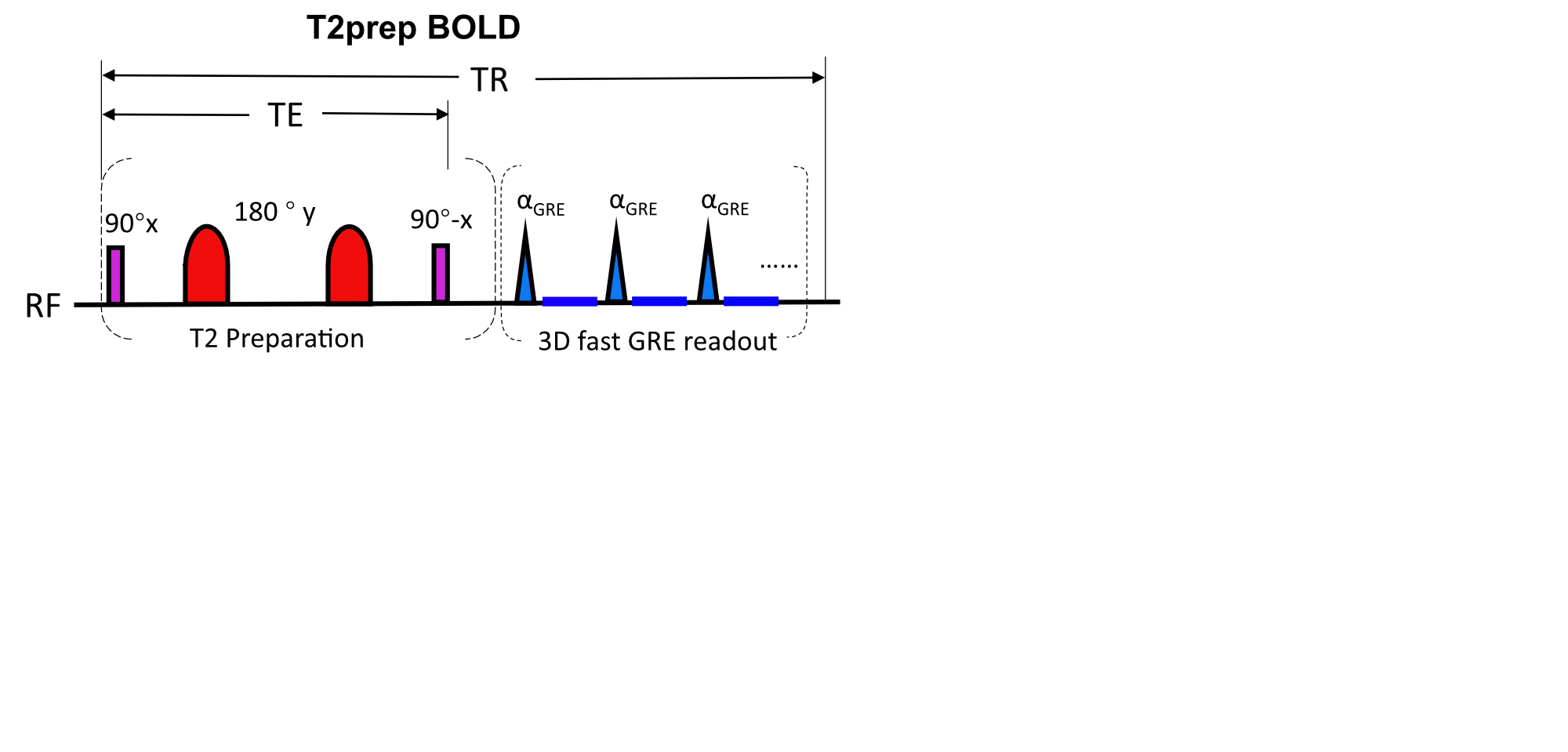
**

**Figure S1.** Pulse sequence diagram of the three-dimensional (3D) T2-prepared (T2prep) blood oxygenation level–dependent (BOLD) functional MRI approach. The T2 prepration module consists of a 90o pulse, followed by two 180o refocusing pulses and a second 90o pulse with an opposite phase (-x) to generate the BOLD contrast. A 3D fast gradient echo (GRE) readout that is commonly used in anatomical MRI is deployed immediately after T2prep. TR: time of repetition for one entire image volume. TE: time of echo. αGRE: flip angle in the 3D fast GRE readout.

**
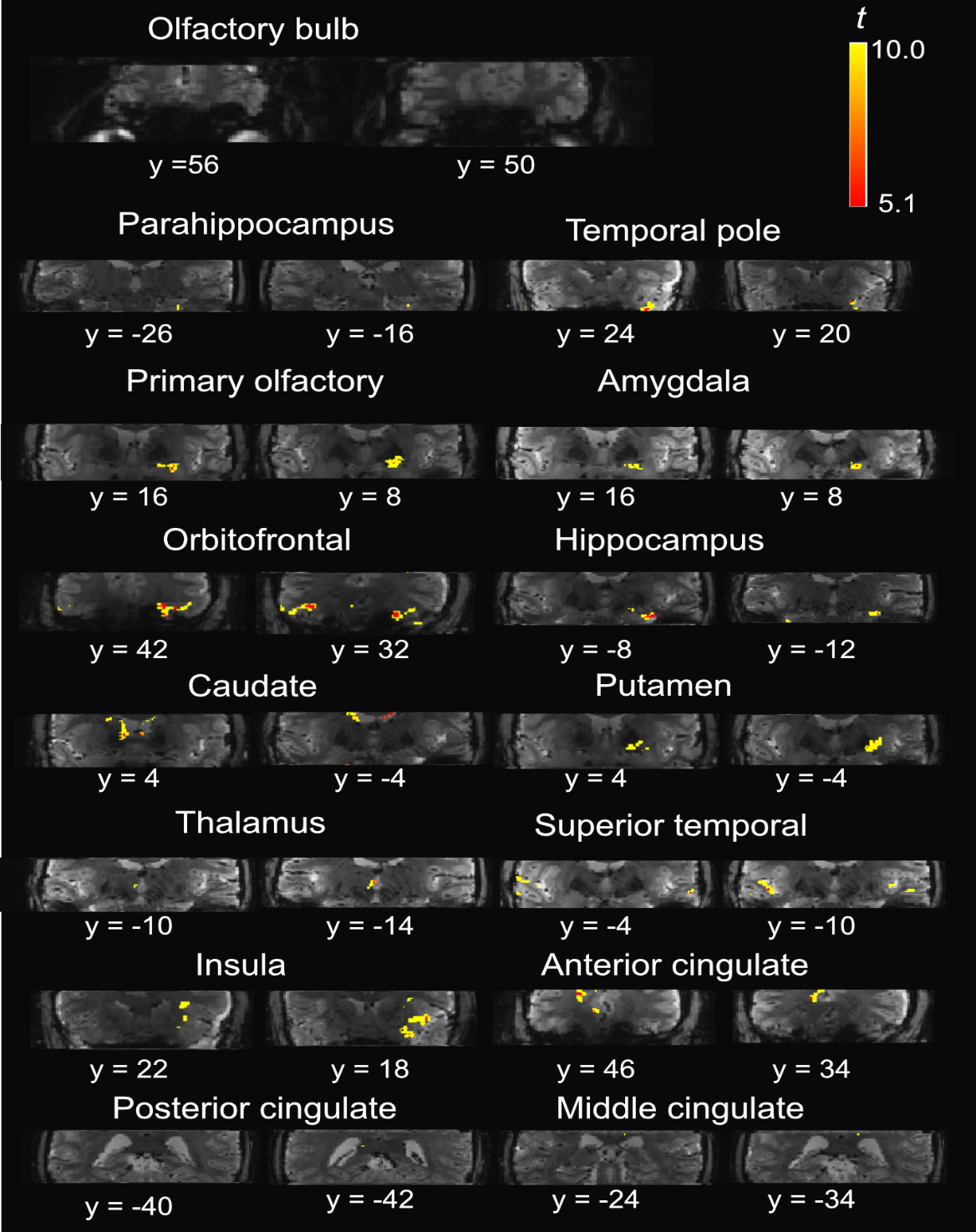
**

**Figure S2.** Representative positive activation maps from GRE EPI BOLD fMRI scans during the olfactory stimulation from one participant. The t-scores of significantly positively activated voxels are overlaid on the GRE EPI BOLD images from the same participant. Two slices are shown for each ROI. But the entire activated cluster in each ROI covered more slices.

**
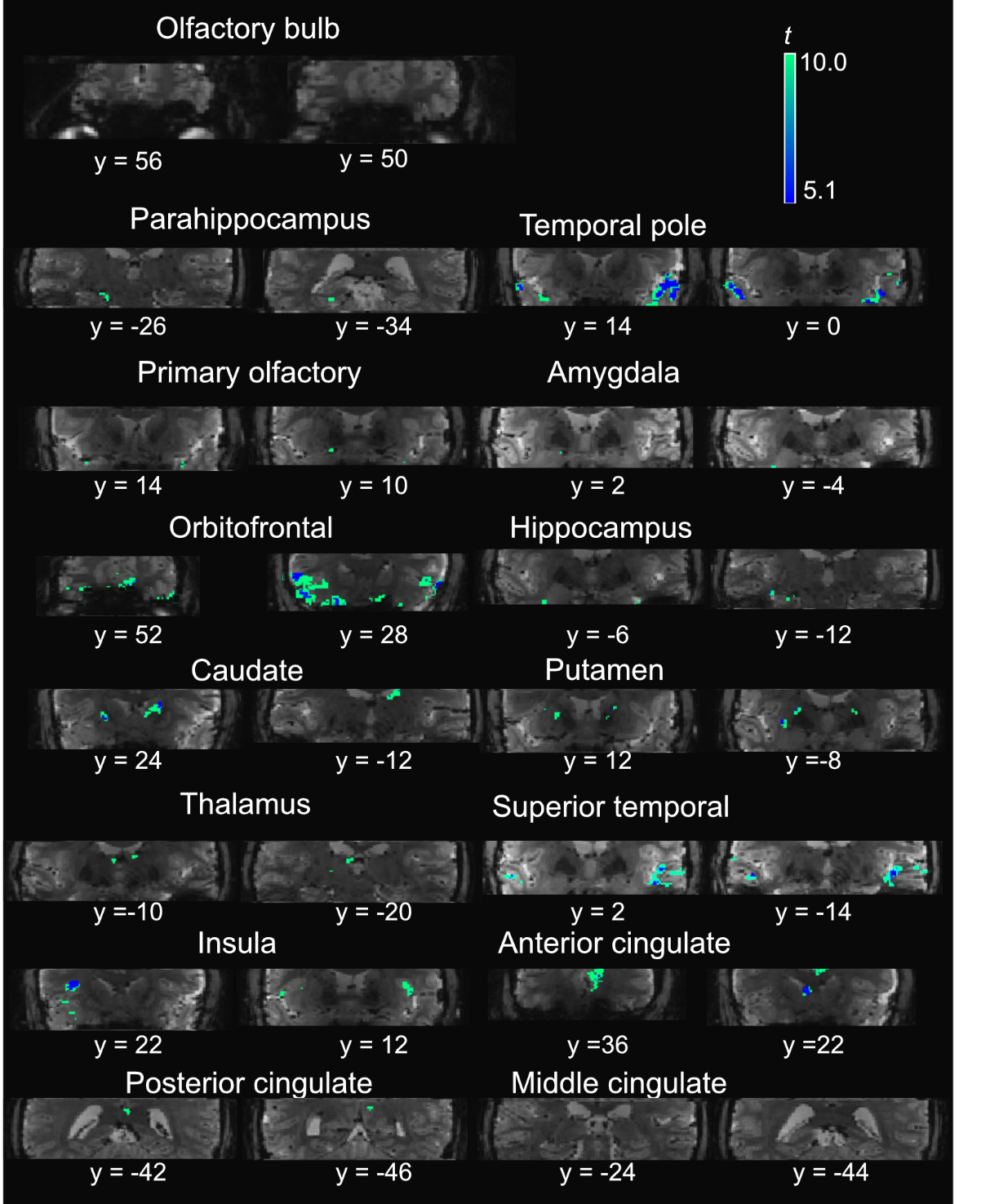
**

**Figure S3.** Representative negative activation maps from GRE EPI BOLD fMRI scans during the olfactory stimulation from one participant. The t-scores of significantly negatively activated voxels are overlaid on the GRE EPI BOLD images from the same participant. Two slices are shown for each ROI. But the entire activated cluster in each ROI covered more slices.


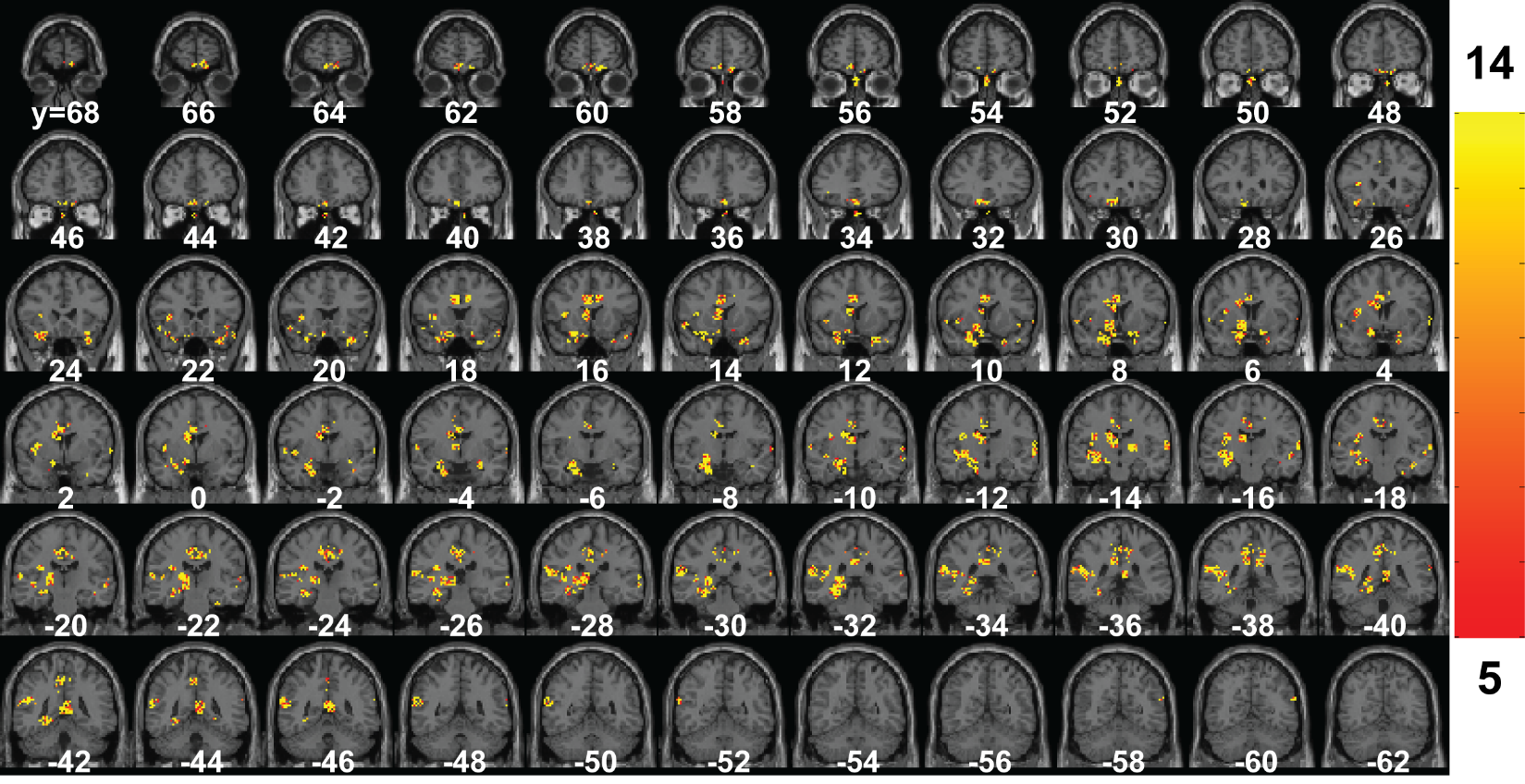


**Figure S4:** Map of positively activated voxels combined from all participants (n = 14). Individual maps were normalized to the Montreal Neurological Institute (MNI) space. The combined map is overlaid on MNI normalized anatomical images. The intensity of each activated (highlighted) voxel represents the number of participants from which the voxel was positively activated. The color bar indicates the range of this number displayed. Only voxels that were activated in at least 5 subjects were included.


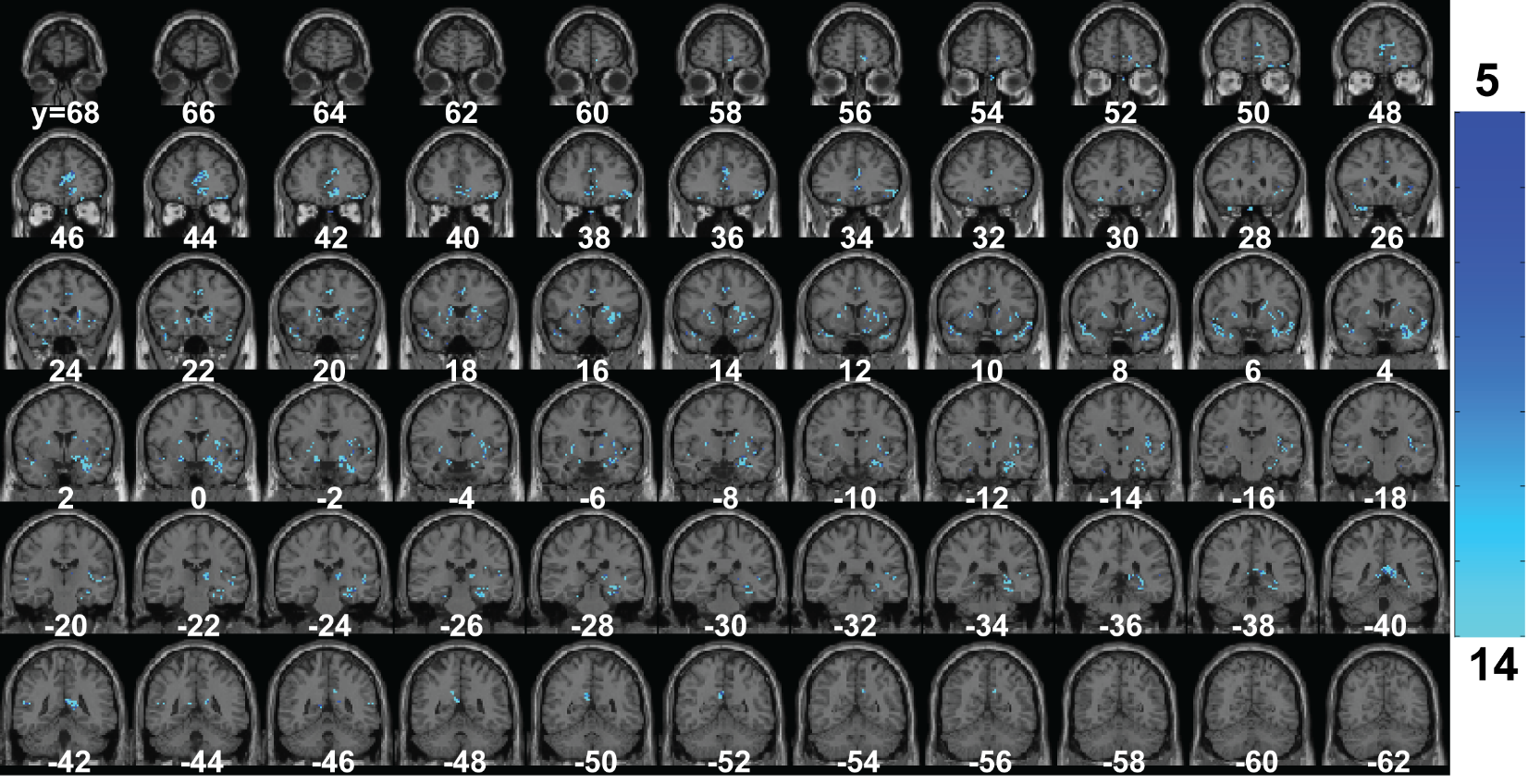


**Figure S5:** Map of negatively activated voxels combined from all participants (n = 14). Individual maps were normalized to the Montreal Neurological Institute (MNI) space. The combined map is overlaid on MNI normalized anatomical images. The intensity of each activated (highlighted) voxel represents the number of participants from which the voxel was negatively activated. The color bar indicates the range of this number displayed. Only voxels that were activated in at least 5 subjects were included.
